# Colonization of Northern Europe by *Zygaena filipendulae* (Lepidoptera)

**DOI:** 10.1002/ece3.5082

**Published:** 2019-04-03

**Authors:** Mika Zagrobelny, Lene Dalsten, Axel Hille

**Affiliations:** ^1^ Department of Plant and Environmental Sciences and Copenhagen Plant Science Centre University of Copenhagen Frederiksberg C Denmark; ^2^ Institute of Applied Statistics Dr Jörg Schnitker Germany

**Keywords:** Bayesian phylogeography, burnet moths, ecological clustering, ice age refugia

## Abstract

Northern and mountainous ice sheets have expanded and contracted many times due to ice ages. Consequently, temperate species have been confined to refugia during the glacial periods wherefrom they have recolonized warming northern habitats between ice ages. In this study, we compare the gene *CYP405A2* between different populations of the common burnet moth *Zygaena filipendulae* from across the Western Palearctic region to illuminate the colonization history of this species. These data show two major clusters of *Z. filipendulae* populations possibly reflecting two different refugial populations during the last ice age. The two types of *Z. filipendulae* only co‐occur in Denmark, Sweden, and Scotland indicating that Northern Europe comprise the hybridization zone where individuals from two different refugia met after the last ice age. Bayesian phylogeographic and ecological clustering analyses show that one cluster probably derives from an Alpe Maritime refugium in Southern France with ancestral expansive tendencies to the British Isles in the west, touching Northern Europe up to Denmark and Sweden, and extending throughout Central Europe into the Balkans, the Peleponnes, and South East Europe. The second cluster encompasses East Anatolia as the source area, from where multiple independent dispersal events to Armenia, to the Alborz mountains in north‐western Iran, and to the Zagros mountains in western Iran are suggested. Consequently, the classical theory of refugia for European temperate species in the Iberian, Italian, and Balkan peninsulas does not fit with the data from *Z. filipendulae *populations*, *which instead support more Northerly, mountainous refugia.

## 1 INTRODUCTION

During the Quaternary time‐period (2.6 million years ago until present), several ice ages (glacial periods) have resulted in expansion and contraction of Northern and mountainous ice sheets. Consequently, temperate species have been confined to refugia during the glacial periods wherefrom they have recolonized warming northern habitats between ice ages (interglacials). In Europe, many temperate species have been shown to inhabit the Iberian, Italian, and Balkan peninsulas during glacials (Hewitt, 2011; Stewart, Lister, Barnes, & Dalen, 2010). Furthermore, there are some indications that cryptic northern refugia, comprising of, for example, deeply incised sheltered mountain valleys, could also have played a role for the survival and recolonization of some temperate species (Schmitt & Varga, 2012). Since glacials are approximately 10 times longer than interglacials, they are thought to have played a major role in population divergence for temperate species (Hewitt, 2011). Studies have shown that populations become extinct as their habitat disappears, rather than physically moving into refugia at the beginning of a glacial period (Hampe & Petit, 2005). Accordingly, populations in long‐term refugia (inhabited for at least one full glacial/interglacial cycle) are descended from populations that were already in place before the onset of glaciation (Hewitt, 2011). Long‐term refugia are therefore expected to harbor the greatest level of genetic diversity within a species range, whereas populations from recent colonizations have the least diversity (Hewitt, 2011). The last glacial period occurred from approximately 110,000 to 12,000 years ago, and Denmark, the rest of Scandinavia and Scotland, was covered in ice until at least 15,000 years ago, resulting in most present day species having arrived in these areas after this time.

Phylogeographic analyses can be used to unravel historical processes resulting in contemporary population distributions based on gene sequences (Hickerson et al., 2010). Bayesian phylogeographic and ecological clustering (BPEC) (Manolopoulou & Hille, 2018; Manolopoulou, Hille, & Emerson, 2019) allows estimation of the posterior probabilities for haplotype tree networks under a coalescent‐based migration–mutation model (Manolopoulou & Emerson, 2012). BPEC combines an evolutionary model for the genealogical relationships among sampled DNA sequences, specified as latitudes and longitudes, together with a geographical model representing splitting and dispersal events forming spatial clusters, assuming that population substructure is the result of individuals migrating to a new geographical area.

**Figure 1:**
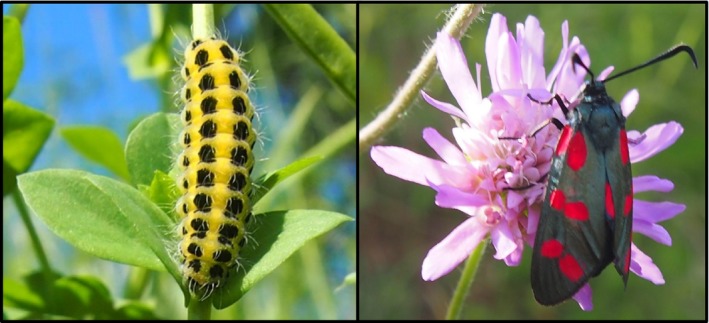
Larvae and adult *Zygaena filipendulae, *the larvae perching on its food plant *Lotus corniculatus*

Burnet moths (Zygaena, Lepidoptera) are brightly colored day flying moths comprising 98 species distributed across most of Europe. *Zygaena filipendulae* (Figure 1) are very common and especially cold‐hardy burnet moths occurring as far North as Northern Norway and Scotland as well as across most of Europe. Hofmann and Tremewan (2017, p. 49) inferred hypothetical areas of origin of the genus *Zygaena* and found clear indications for a primary differentiation of the species groups belonging to the “*filipendulae*”—stem group from the Balkans, around the Black Sea, and Caucasus region, from where several species expanded during different epochs. *Zygaena filipendulae* was stated to have a Euro–Siberian distribution belonging to those species groups derived from the Middle East. Although Zygaena moths normally do not disperse very far (<1–2 km) (Tremewan, 2006), rapid colonizations of suitable habitats have been observed for *Z. filipendulae* in Denmark (M. Zagrobelny, unpublished). When the preferred larval food plant *Lotus corniculatus* (Zagrobelny, Bak, & Møller, 2008) is sown, for example along the bank of a newly built or renovated road, *Z. filipendulae* can appear within a few years.

**Figure 2:**
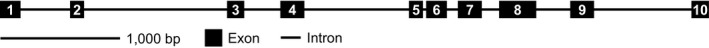
Intron‐Exon organization of CYP405A2 in *Zygaena filipemdulae*

*Zygaena filipendulae* has been established as a model system to study the evolution and roles of its chemical defense compounds, the cyanogenic glucosides linamarin and lotaustralin (Zagrobelny & Møller, 2011; Zagrobelny et al., 2014). The biosynthetic and degradative pathways of these compounds have been solved in *Z. filipendulae *(Jensen et al., 2011; Pentzold et al., 2017), and the first enzyme in the biosynthetic pathway, *CYP405A2*, was used for the present study (Figure 2). *CYP405A2* is ~6,000 bp long with nine introns ranging from 79 to 1,278 bp in length. Accordingly, ~4,500 bp of the gene is noncoding introns. Such noncoding nuclear regions of a genome have previously been shown to be optimal for studies of populations from a species distribution range, due to the rapid accumulation of variation within them, in contrast to the low level of variation accumulating in coding regions (Hewitt, 2011).

In this study, we compare the coding and noncoding parts of *CYP405A2* genes from different populations of *Z. filipendulae* from across the Western Palearctic region to show the pattern of colonization of this species. BPEC analyses are used on the more conservatively evolving coding parts of *CYP405A2* to identify genetically distinct geographical population clusters and ancestral locations in *Z. filipendulae*.

## 2 MATERIALS AND METHODS

### 2.1 Biological specimens and sequencing

*CYP405A2* was sequenced and compared for 1–2 specimens from 30 different populations of *Z. filipendulae*. Larvae or moths were collected from seven populations around Denmark, and *Z. filipendulae* moths from a further 23 populations were obtained from collaborators around Europe (Table 1) (see *Acknowledgments*) either preserved in ethanol or as dried specimens. Genomic DNA was isolated from moths or larvae using DNeasy Blood and Tissue kit (Qiagen 69504). *CYP405A*2 genes from the specimens were PCR amplified from genomic DNA diluted to a concentration of 10 ng/µl, using the following program: denaturation 94°C for 2 min followed by 30 rounds of denaturation at 94°C for 30 s, annealing at 60°C for 30 s, and extension at 72°C for 1 min. Final extension was at 72°C for 7 min. Primers are shown in Supporting Information Table S1. Three different polymerases were used in the PCR reactions: proofreading X7 (made in our laboratory) or nonproofreading TaKaRa (TaKaRa Bio Company) or KapaTaq (KapaBioSystem KR0352). The PCR products were purified on 1% agarose gels and extracted with a gel extraction kit (Omega E.Z.N.A Gel Extraction Kit D2500‐02). The purified fragments were ligated into commercially available vectors: X7 products into pJET1.2 (CloneJet K1232), the other two into pCR2.1TOPO TA (Invitrogen 450641). The ligation mixture was then transformed into either E.Cloni (Lucigen) or TOP10 (Invitrogen) cells and plated on either ampicillin plates (pJET1.2) or Kanamycin plates (pCR2.1TOPO). Selected colonies were grown overnight, and the extracted plasmid DNA was sequenced (Macrogen Europe).

**Table 1:** Populations of *Zygaena filipendulae* analyzed in this study

Location number	Population	Coordinates	
12 (I) 16 (II)	Zf Kvistgård (Denmark)	N56°0′7.98″ 56.002217	E12°30′47.28″ 12.513133
31 (I) 32 (II)	Zf Citytwo (Denmark)	N55°38′4.62″ 55.634617	E12°15′44.88″ 12.262467
19 (II) 30 (I)	Zf Amager (Denmark)	N55°37′45.12″ 55.6292	E12°34′54.6″ 12.581833
20 (II) 29 (I)	Zf DTU (Denmark)	N55°47′21.9″ 55.789417	E12°30′28.32″ 12.507867
5 (I) 34 (II)	Zf Møn (Denmark)	N54°57′39.6″ 54.961	E12°30′33.66″ 12.50935
6 (I) 37 (II)	Zf SDU (Denmark)	N55°22.17667 55.369611	E10°25.30167 10.42169
18 (I) 35 (II)	Zf Anholt (Denmark)	N56°42 56.7	E11°33 11.55
25 (a) 26 (b)	Zf Sweden	N58°26′48.725″ 58.446868	E11°26′33.994″ 11.442776
14 (I) 24 (II)	Zf Scotland East	N57°18′42.998″ 57.311944	E1°59′47″ −1.996389
1 (I) 36 (II)	Zf Scotland West	N57°51′58.118″ 57.866144	E5°14′24.612″ −5.24017
4	Zf England Early	N50°09′48″ 50.163333	W5°40′15″ −5.670833
3	Zf England Late	N50°12′37.9″ 50.210528	W5°24′27.6″ −5.407667
17	Zf France Northeast	N49°6′24.75″ 49.106875	E6°4′32.333″ 6.075648
23 (I) 27 (II)	Zf France Southeast	N44°24′56.88″ 44.4158	E5°40′59.88″ 5.6833
2	Zf France West	N47°13′37.2″ 47.227	E0°1′30″ 0.025
11 (I) 33 (II)	Zf Switzerland	N46°20′30.588″ 46.34183	E7°0′41.436″ 7.01151
43	Zf Pyrenees	N42°43′23.484″ 42.72319	E0°7′4.534″ 0.117926
7	Zf Italy North	N46°36′2.999″ 46.600833	E11°34′32.002″ 11.575556
10	Zf Italy South	N41°26′30.001″ 41.441667	E13°28′32.002″ 13.475556
13	Zf Greece North	N41°18′4″ 41.301111	E23°54′51.001″ 23.914167
22	Zf Greece South	N37°54′24.998″ 37.906944	E22°4′14.002″ 22.070556
8	Zf Poland West	N51°44′36.24″ 51.7434	E15°35′47.278″ 15.596466
15	Zf Poland Middle	N51°18′52.999″ 51.314722	E21°55′25.619″ 21.923783
9	Zf Poland Southeast	N49°6′56.045″ 49.115568	E22°42′16.632″ 22.70462
21	Zf Poland South	N49°50′51.72″ 49.8477	E18°35′25.86″ 18.590517
28	Zf Turkey West	N36°0′36″ 36.01	E36°1′12″ 36.02
38	Zf Turkey East	N37°33′24.43″ 37.556786	E43°56′57.786″ 43.949385
41 (a) 42 (b)	Zf Armenia	N40°41′3.883″ 40.684412	E44°57′45.587″ 44.962663
40	Zf Iran Northwest	N36°1′56.701″ 36.804057	E45°55′43.198″ 49.136548
39	Zf Iran Northeast	N36°48′14.605″ 36.032417	E49°8′11.573″ 45.928666

### 2.2 Assembly and phylogenetic analysis

**Figure 3:**
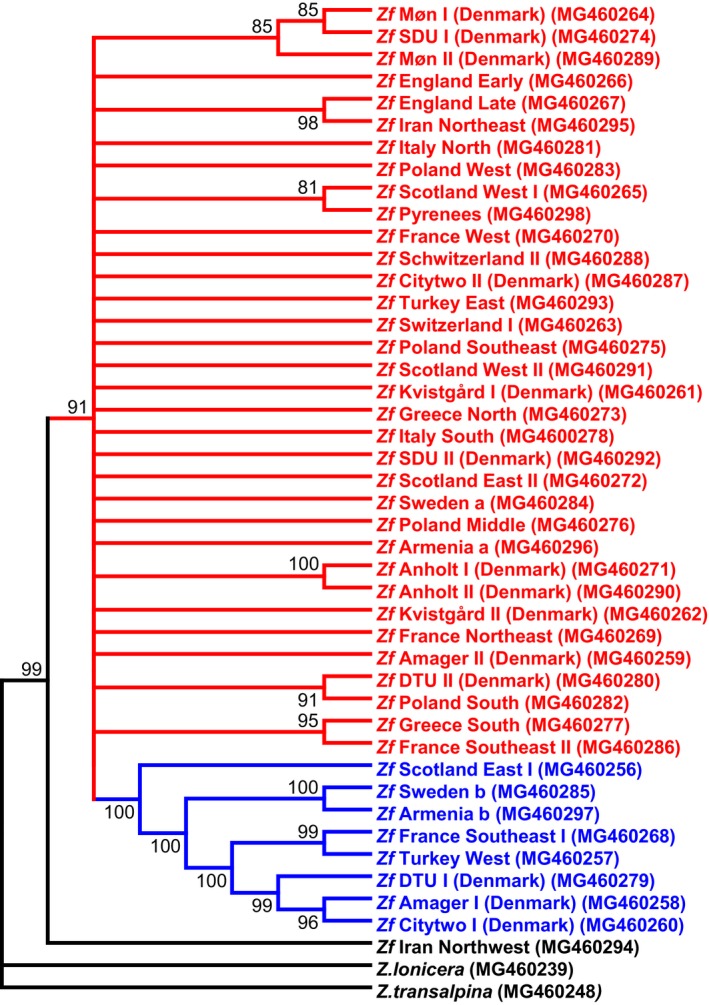
Phylogenetic tree constructed with the maximum‐likelihood method and the Tamura 3‐parameter model with a discrete Gamma distribution and allowing for invariant sites. A total of 1,000 bootstrap replicates were carried out, and the tree is condensed to show only branches with a bootstrap above 70. The Western‐Central‐Northern European cluster is shown in red and the Caucasian–Iranian cluster is shown in blue

The fragments of CYP405A2 obtained from all the specimens were manually assembled into full‐length genomic sequences in MEGA7 (Kumar, Stecher, & Tamura, 2016). They were aligned partly by MUSCLE (Edgar, 2004a,2004b) and partly manually (due to the difficulty of aligning intron sequence in any computer program). Genes were submitted to Genbank (Accession numbers shown on Figure 3). The evolutionary history was inferred with the maximum‐likelihood method based on the Tamura 3‐parameter model and a discrete Gamma distribution. This model was chosen based on the model test from the MEGA7 program, where the model with the lowest BIC score (Bayesian Information Criterion) is considered to describe the observed substitution pattern best. Phylogenetic trees were also constructed using BEAST (Suchard et al., 2018) with essentially the same results as MEGA7 (results not shown). Evolutionary distances, ancestral sequences, and molecular clock analyses were all carried out in MEGA7 with default parameter settings.

### 2.3 Bayesian phylogeographic and ecological clustering analyses

Bayesian phylogeographic and ecological clustering was used to identify genetically distinct geographical population clusters and ancestral locations in *Z. filipendulae*. BPEC relies on parsimony in order to reduce the number of candidate trees. Each possible tree defines a set of possible migration events that may have led to the observed population substructure. Different haplotype trees and migration events are explored through Markov chain Monte Carlo (MCMC) sampler calculating posterior probabilities in a fully model‐based framework, thus controlling for uncertainty in haplotype relationships. A total of 43 concatenated exon sequences of CYP405A2 (1,467 bp) were available for the Bayesian phylogeographic clustering analysis. Haplotype reconstruction used 42 distinct haplotypes with 67 parsimony informative sites and further (178 ‐ 42) intermediate haplotypes for the most parsimonious haplotype network. The analysis was run with two Markov chain Monte Carlo initializations for 2,000,000 iterations each with 2,000 steps saved, taking the strict parsimony level option at minimum, ds = 0. The dimensionality of the data was *p* = 2 for geographical dimensions longitude and latitude. The posterior mass for the number of clusters strongly concentrates around 2, with the posterior probability of two clusters being >0.81. The two clusters are designated as the “Western‐Central‐Northern European” *Z. filipendulae* (European) and “Caucasian–Iranian” *Z. filipendulae* cluster (Caucasian), respectively. The root location is the same regardless if BPEC analyses are run with or without recombinant sites.

## 3 RESULTS

To unravel the relationships between different populations of *Z. filipendulae*, CYP405A2 sequences from 43 specimens were analyzed. *Z. filipendulae* *CYP405A2* sequences are between 92% and 99% identical to each other (Table 2) and the outgroups *Zygaena lonicera* and *Zygaena transalpina* are 89%–93% and 82%–85% identical to them, respectively. From the phylogenetic tree (Figure 3), it is evident that the specimens largely fall into two clusters (the European cluster and the Caucasian cluster). The specimen from Iran Northwest seems ancestral to both clusters. Remarkably, some populations (from Denmark, Scotland East, and France Southeast) have specimens from both clusters and the single specimens analyzed from Sweden and Armenia are both heterozygous with one allele belonging to each cluster. The longest evolutionary distances between any of the specimens analyzed (Table 2) can be found between members of the European cluster and the Caucasian cluster.

**Table 2:** Evolutionary distances within clusters and populations of *Zygaena filipendulae*

Within European cluster	0.01–0.03
Within Caucasian cluster	0.01–0.05
Between European and Caucasian clusters	0.03–0.07
Anholt	0.00
Møn	0.00
SDU	0.01
Kvistgård	0.02
Scotland West	0.01
Switzerland	0.02
Scotland East	0.03
Sweden	0.03
Armenia	0.04
France Southeast	0.05
DTU	0.05
Amager	0.05
City 2	0.06

More variation is evident within the Caucasian cluster than within the European cluster (Table 2), and in some European populations, there is almost no variation between specimens analyzed. When looking more closely at the introns, it is clear that most of the variation can be found in intron 1, 2, and 4, whereas intron 5–9 are a lot more conserved. The large variation in intron 4 is the main reason for the segregation of the phylogenetic tree into the Caucasian and European cluster, since there are two “versions” of this intron, the Caucasian (631 bp ± 10) and the European (971 bp ± 33). When calculating an ancestral sequence for the Caucasian and European cluster as well as an ancestor of all *Z. filipendulae* populations, the ancestors both have features mostly from the European cluster in intron 4. They both group with Greece South and France Southeast in a maximum‐likelihood tree with 1,000 bootstrap replicates (data not shown). The accuracy scores for the ancestral sequences are 0.998 and 0.997, respectively, implying that on average any base has a 99.8% or 99.7% chance of being correct (Hall, 2011). This indicates that intron 4 has evolved from a European common ancestor in spite of more variation present in the Caucasian cluster.

**Figure 4:**
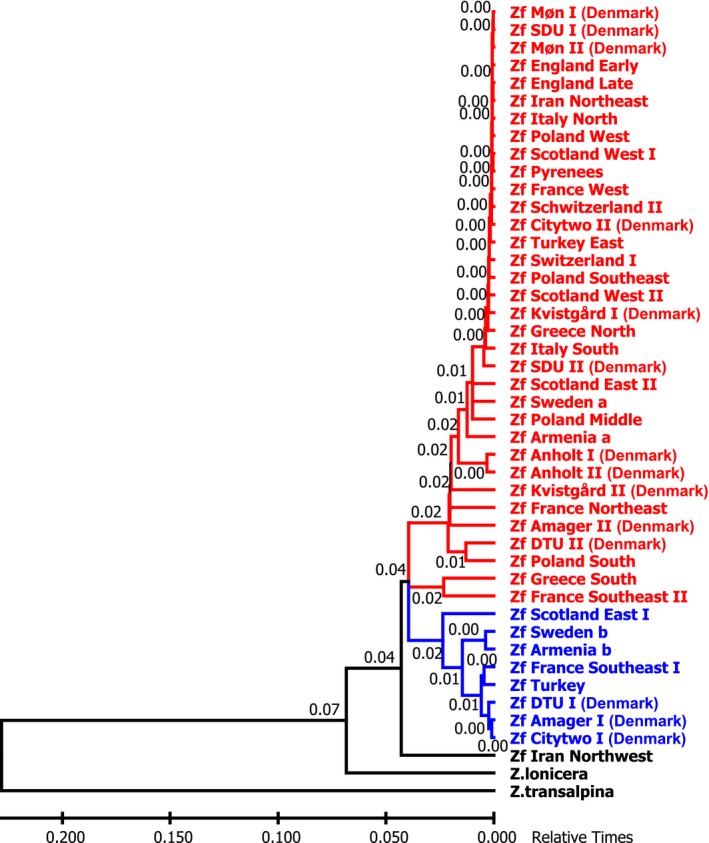
Phylogenetic tree with relative times inferred using the maximum‐likelihood method under the Tamura 3‐parameter model. The rates among sites were treated as a Gamma distribution with invariant sites. There were a total of 8,248 positions in the final dataset. The Western‐Central‐Northern European cluster is shown in red and the Caucasian–Iranian cluster is shown in blue

Since Tajimas rate test and molecular clock analysis using maximum likelihood showed equal evolutionary rate throughout the tree, it is possible to do a molecular clock analysis. However, a fixed time point is needed to calibrate the clock, and the paleontological record only contain two different Zygaenidae genera described from the Miocene (5–23 MYA) in central Europe (Naumann, 1987). None of them can be ascribed to a specific species; one is probably an early Epizygaenella (sister group to Zygaena) and the other could be either Praezygaena, Reissita, Epizygaenella, or Zygaena. A *Z. filipendulae* + *Z. transalpina* + *Zygaena nevadensis* stem group was previously established based on molecular‐phylogenetic reasoning (Niehuis, Hofmann, Naumann, & Misof, 2007), but to date there is no evidence of when *Z. filipendulae* diverged from other Zygaena species. Consequently, time points based on available habitat after the withdrawal of the Northern European ice sheets were used to calibrate the molecular clock. Since Scotland East is the earliest diverging species in the Caucasian cluster, this divergence point could presumably not be earlier than 15,000 years ago (http://www.donsmaps.com/icemaps.html), because Scotland was covered in ice before that timepoint. When using this calibration point, timings of other events in the phylogenetic tree appeared to have happened later than they logically could have, for example *Z. lonicera* should have split from *Z. filipendulae* only 22,000 years ago which seems highly unlikely. Consequently, the split between the European and the Caucasian cluster appear to be much earlier than the 15,000 years ago used to calibrate the tree and consequently, it is not possible to assign a reliable calibration time to the molecular clock tree (Figure 4). As evident in Figure 4, most of the European populations have diverged from each other quite recently, indicating extensive interbreeding between European *Z. filipendulae* populations.

**Figure 5:**
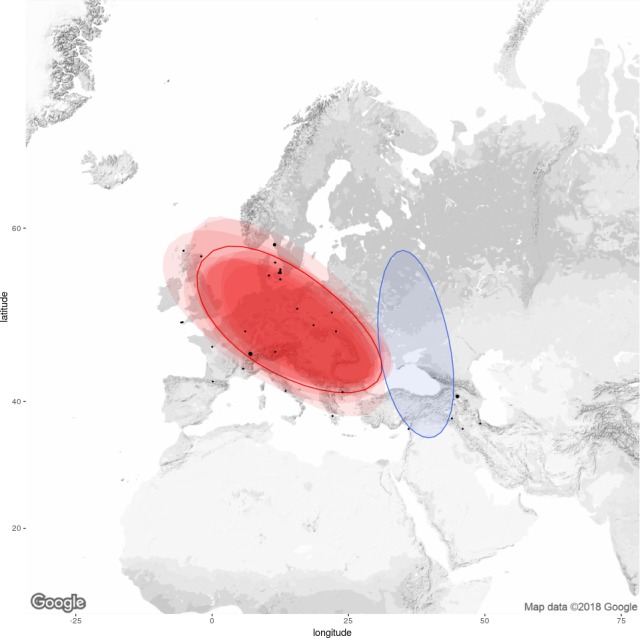
Map of the Wester Palearctic region with two phylogeographic clusters of *Zygaena filipendulae* assigned as a colored contour plot. The contour plots are centered at the “center” of each population cluster, and the colored regions show the radius of 50% concentration contours around it. Bigger black dots show the ancestral locations with the highest posterior probability for each location. Locations situated beyond the clusters could also belong to these clusters, but with low probability

**Figure 6:**
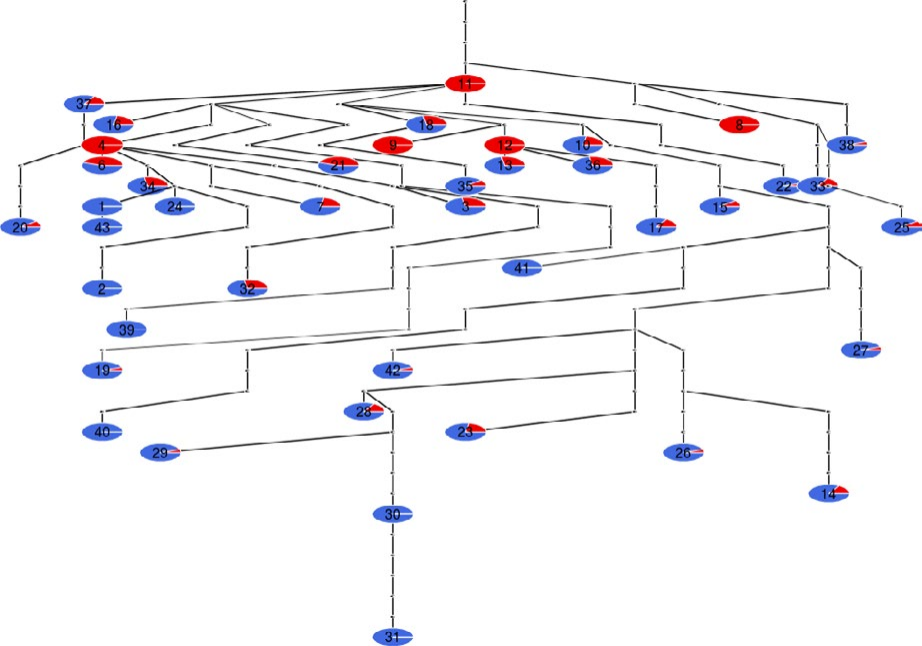
Schematic representation of the unique maximum a posteriori estimate (MAP) of the haplotype clustering. Color corresponds to cluster membership probability and size of node to the number of individuals sampled with each sequence. Edges represent single effective mutations and black dots represent unobserved intermediate haplotypes. In this case, all edges have effectively no posterior uncertainty under the model, so they all appear with equal thickness

Haplotypes of *Z. filipendulae* were assigned to two phylogeographic clusters with high posterior probabilities (0.81, 0.19) (Figure 5) by BPEC. The root node of the haplotype network was inferred as missing, possibly due to no sampling from the respective region, or likewise probable, the signal for finding the root in the network was too low. Sites with the highest posterior probabilities as ancestral population locations were sites 11, 41, and 25 (7.8%, 6.5%, 6.5%): Location 11 near Genfer See, Switzerland is connected through the Gateway of Burgundy to the Maritime Alps in Southern France, a potential glacial refugium. Location 41, north of Lake Sevan, Armenia, has an unhampered connection to the Black Sea coast, and/or to the Caucasus/Southern Caspian region, both important glacial refugia. Location 25, 90 km north of Göteborg along the Skagerrak coastline in Sweden, must be a haplotype that is a relict of a former recolonization from the southern route out of Central and Northern Europe (Figures 5 and 6, Table 1).

High posterior assignment probabilities to the European cluster were obtained for the following haplotypes (≥0.95): 1 Scotland West, 2 France West, 24 Scotland East II, 30 Amager I, 31 Citytwo I, 39 Iran Northeast, 40 Iran Northwest, 41 Armenia a, 43 Pyrenees. High posterior assignment probabilities to the “Caucasian–Iranian” cluster (Caucasian) were obtained for the following haplotypes (≥0.95): 4 England Early, 8 Poland West, 9 Poland Southeast, 11 Switzerland I, 12 Kvistgaard I. The remaining haplotypes share origins from both clusters in varying proportions, but usually with higher probabilities to the West‐Central‐North cluster (≥0.67), or in two cases assigned with equal probability to both clusters (0.50) (6 SDU I and 21 Poland South). Haplotypes 6 and 21 both had similarly low probabilities for two alternative clusters (posterior probabilities of 0.5), and so could not be unambiguously assigned to a single cluster. It is remarkable that in specimens tested from two neighboring populations in England, only differing in the timing of the end of diapause in spring (England Early and England Late), we found a 100% assignment to the Caucasian cluster for the early specimen, and a two‐third assignment to the European population for the late specimen. Another example is the haplotypes of both strands of the gene in the Armenian specimen, being assigned with high proportion to the predominant West‐Central‐North cluster though located in the Eastern region.

Theoretical predictions of coalescent theory states that a root haplotype is expected to have a higher number of haplotype connections in the network, rather than being close to the tips. The fact that BPEC does provide a finite (probability) distribution of haplotype trees—as well as the existence of the underlying migration model—allows this method to incorporate the uncertainty in the haplotype rooting.

## 4 DISCUSSION

We analyzed specimens from populations of *Z. filipendulae* across the Western Palearctic region to infer their phylogeographic relationships. Only a fraction of the total genetic diversity within populations of a species can be seen in any study, relying on the number of specimens and populations analyzed. Most previous studies have relied on the mitochondrial COI gene and a sequence of 648 bp in length (Huemer, Mutanen, Sefc, & Hebert, 2014; Mutanen et al., 2012). We sequenced ~6,000 bp from each specimen, and the results clearly show two population clusters, shown in red (West‐Central‐North Europe) and blue (Caucasian–Iranian) derived from an unknown ancestor. The clusters refer to the two polydynamic lineages of *Z. filipendulae*, probably derived from one refugium in the Alpe Maritime in Southern France (European) and another in East Anatolia (Caucasian). The Alpe Maritime refugium has ancestral expansive tendencies to the British Isles in the west, touching Northern Europe up to Denmark and Sweden, and extending throughout Central Europe into the Balkans, the Peloponnese and South East Europe. The second cluster encompasses East Anatolia (Caucasian) as the source area, from where multiple independent dispersal events to Armenia, to the Alborz mountains in north‐western Iran, and to the Zagros mountains in western Iran are suggested (Figure 5). Subsequently, we found elongated and directed expansion to the East of Europe right to the Balticum, corroborating the fact that the Swedish specimen (both haplotypes) shows traces of an old colonization from the Caucasian–Iranian route indicated by the one‐third eastern origin. Lower latitudes and varied topography make the southern Caucasus, like southern Europe and northern Turkey, suitable places for multiple glacial refugia where genetic lineages may have diverged over several ice ages (Seddon, Santucci, Reeve, & Hewitt, 2002).

The phylogenetic tree suggests that the two clusters of *Z. filipendulae* only co‐occur in a few populations, mainly in Denmark, Sweden, and Scotland indicating that Northern Europe comprise the hybridization zone where individuals from the two different refugia met after the last ice age. Based on molecular clock analyses, the common ancestor of all contemporary specimens is quite a bit older than the end of the last ice age, suggesting that splits between populations originate in an earlier ice age or other habitat limiting event. The calculated ancestral sequence for the European and the Caucasian cluster has more features from the European cluster than the Caucasian cluster, although more variation within the Caucasian cluster was evident. This could signify that the European cluster is more ancient, but more interbreeding within the European populations has taken place, while the Caucasian population has been more isolated. The low amount of variation between specimens from island populations (Anholt and Møn) could indicate recent colonization events, as seen for the Carabid beetle *Carabus arcensis* on other Danish islands (Hansen, Justesen, Olsen, & Solodovnikov, 2017), but could also be a sign of inbreeding or an evolutionary sweep.

The existence of contemporary Nordic populations of cold‐hardy species is normally explained by colonization after the last ice age from southern, western, and/or eastern refugia. However, recent studies suggest that in situ or close‐by survival of at least some insect and plant species was possible during the last ice age (Quinzin, Normand, Dellicour, Svenning, & Mardulyn, 2017; Simonsen & Huemer, 2014). One notable example is the leaf beetle *Gonioctena intermedia* which was suggested to have survived in Southern Norway and Northern Central Europe quite close to the ice sheets together with its food plants *Prunus padus* and *Sorbus aucuparia* even during the last glacial maximum. The current genetic diversity of this insect species seem to have been shaped by a combination of survival in northern Europe combined with postglacial colonization of the remaining current range from central European refugia perhaps in the Alps (Quinzin et al., 2017). A similar scenario could explain the results from the present study, where *Z. filipendulae* populations split from a common ancestor much earlier than previously thought and then survived one or more ice ages in several cryptic northerly refugia.

The two English *Z. filipendulae* populations sampled in this study have very different life histories since one population emerge from diapause one month earlier than the other in the spring and consequently also appear as adults one month earlier (G. Tremewan, personal communication). Despite these differences, both populations are very closely related in the phylogenetic tree, although the BPEC analyses showed them to mostly belong to the two different clusters. The two Scottish populations are quite different from each other, one of them very similar to the English populations while the other is in the Caucasian cluster. This could signify that this population has been isolated since the initial colonization of Great Britain, while the other three British populations have been interbreeding to some extent.

Since insects cannot colonize new areas prior to the establishment of their food plant, it would have been ideal to include *L. corniculatus* in this study. However, *L. corniculatus* is unfortunately routinely sown out along, for example, road banks all over Europe, and the seeds all originate from similar populations. Therefore, it is highly problematic to find enough samples of the “native” plant populations and to avoid dilution of the phylogenetic signal by sequences from the sown plants.

Conclusively, two major groupings of *Z. filipendulae* populations were found in this study which could reflect two different refugial populations during the last ice age, an Alpine/Maritime refugium in Southern France and another in East Anatolia. These two types of *Z. filipendulae* are only co‐occurring in Denmark, Sweden, and Scotland which could indicate that Northern Europe comprise the “hybridization zone” where individuals from the two different refugia met after the last ice age. BPEC analyses identified genetically distinct geographical population clusters and ancestral locations in *Z. filipendulae *and suggest an Alpine/Maritime refugium in Southern France and another in East Anatolia. Consequently, the classical theory of refugia for European temperate species in the Iberian, Italian, and Balkan peninsulas does not fit with the data from *Z. filipendulae *populations*, *which instead support more Northerly, mountainous refugia.

## CONFLICT OF INTEREST

The authors have no conflict of interest to declare.

## AUTHOR CONTRIBUTIONS

MZ conceived and designed the overall study. LD extracted DNA and sequenced genes from specimens. MZ assembled and aligned genes and produced phylogenetic trees. AH did Bayesian phylogeographic analyses. MZ and AH wrote the manuscript, and all authors revised the manuscript.

## Data Availability

DNA sequences: Genbank accessions MG460239, MG460248, MG460256‐MG460298.
